# Fast multiplex real-time PCR assay for simultaneous detection of dog and human blood and *Leishmania* parasites in sand flies

**DOI:** 10.1186/s13071-020-3994-6

**Published:** 2020-04-21

**Authors:** Kamila Gaudêncio da Silva Sales, Débora Elienai de Oliveira Miranda, Marcelo Henrique Santos Paiva, Luciana Aguiar Figueredo, Domenico Otranto, Filipe Dantas-Torres

**Affiliations:** 1 Department of Immunology, Aggeu Magalhães Institute, Oswaldo Cruz Foundation (Fiocruz), Recife, Brazil; 2 Federal University of Pernambuco (UFPE), Caruaru, Brazil; 3 Department of Entomology, Aggeu Magalhães Institute, Oswaldo Cruz Foundation (Fiocruz), Recife, Brazil; 4 Department of Veterinary Medicine, Università degli Studi di Bari, Valenzano, Bari, Italy; 5 Faculty of Veterinary Sciences, Bu-Ali Sina University, Hamedan, Iran

**Keywords:** Phlebotomine sand flies, Blood meal, Brazil, Real-time PCR

## Abstract

**Background:**

The blood-feeding behaviour of female sand flies may increase their likelihood of acquiring and transmitting *Leishmania* parasites. Studies on the host usage by these insects may thus improve our understanding of the *Leishmania* transmission risk in leishmaniasis-endemic areas. Here, we developed a fast multiplex real-time PCR assay for simultaneous detection of dog, human and *Leishmania* DNA in sand flies.

**Methods:**

Primers and TaqMan probes targeting the mitochondrial cytochrome *c* oxidase subunit 1 and cytochrome *b* genes of dog and human, respectively, were combined in a multiplex assay, which also includes primers and a TaqMan probe targeting the *Leishmania* minicircle kinetoplast DNA.

**Results:**

The multiplex assay was 100% specific, with analytical sensitivities of 10^3^ fg/reaction for dog and human and 1 fg for *Leishmania*. By testing field-collected engorged female sand flies (95 *Migonemyia migonei* and two *Nyssomyia intermedia*), 50 *M. migonei* were positive for one or two targets (positivity rates: 45.4% for human, 4.1% for dog and 12.4% for *Leishmania* DNA).

**Conclusions:**

This multiplex real-time PCR assay represents a novel fast assay for detecting dog, human and *Leishmania* DNA in female sand flies and therefore a tool for assessing the risk of *Leishmania* transmission to these hosts in areas of active transmission. 
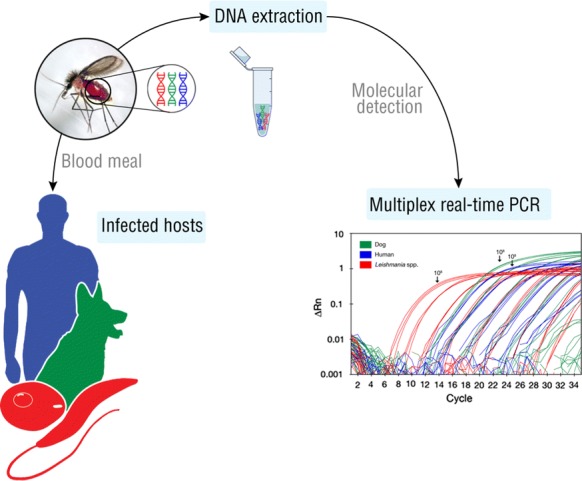

## Background

Sand flies (Psychodidae: Phlebotominae) are insects of paramount medical and veterinary significance, mainly due to their competence in transmitting pathogens to many animal species, including humans [1]. Plant-derived carbohydrates (e.g. nectar, honeydew and phloem sap) are part of the diet of both male and female sand flies, being an essential source of energy for various activities, including flight and reproduction [2]. However, adult females are also blood-feeders and require vertebrate blood as a source of protein for oogenesis [3]. Thus, the feeding behaviour of female sand flies may influence their likelihood of acquiring and transmitting pathogens, including *Leishmania* parasites (Kinetoplastida: Trypanosomatidae), the causative agents of leishmaniases. To date, over 1000 sand fly species have been identified globally, of which more than 50% are exclusively found in the Neotropics [4]. About 98 species of sand flies have been listed as proven or suspected vectors of *Leishmania* spp. [1].

Leishmaniases are among the top ten neglected tropical diseases causing high levels of morbidity and mortality in endemic areas, mainly in tropical and subtropical regions of the world [5]. Brazil, India, Bangladesh, Sudan, South Sudan and Ethiopia concentrate 90% of the global incidence of visceral leishmaniasis (VL), whereas Afghanistan, Algeria, Colombia, Brazil, Iran, Syria, Ethiopia, Sudan, Costa Rica and Peru concentrate ~ 75% of the global incidence of cutaneous leishmaniasis (CL) [5].

An important factor influencing the dynamics of *Leishmania* parasite transmission in endemic areas is the feeding behaviour of female sand flies. For example, to be considered a ‘good vector’ for zoonotic *Leishmania* parasites, females from a given sand fly species should feed frequently on the reservoir host(s) and on humans as well. Therefore, investigations of the blood meals of various species of sand flies are crucial towards a better assessment of the risk of *Leishmania* transmission in areas where leishmaniasis are endemic. Several methodologies to identify blood meal in sand flies have been used, including enzyme-linked immunosorbent assay (ELISA), mass spectrometry, precipitin test and polymerase chain reaction (PCR) [6–11]. More recently, quantitative real-time PCR [12, 13] and PCR followed by amplicon sequencing [14] demonstrated promising results, with high-level sensitivity. Although all these methods generated important information about the feeding behaviour of sand flies, they may present several drawbacks, such as low sensitivity and specificity (e.g. ELISA and precipitin test) and high cost (e.g. mass spectrometry and PCR followed by amplicon sequencing) [12, 15, 16].

In this context, we developed a fast multiplex real-time PCR assay for simultaneous detection of dog and human blood meals and *Leishmania* parasites in sand flies, with high analytical sensitivity and specificity, as well as relatively low cost.

## Methods

### Blood samples and *Leishmania* parasites

Venous blood samples (2 ml) were withdrawn from a dog and a human in EDTA tubes (Vacuette K3EDTA tube; Greiner Bio-One, Kremsmünster, Austria) and frozen at − 20 °C until DNA extraction. Reference strains of *Leishmania infantum* (MHOM/BR/76/M4192) and *Leishmania braziliensis* (MHOM/BR/1975/M2903) were obtained from the Leishmaniasis Reference Service of the Aggeu Magalhães Institute (Fiocruz-PE, Recife, Brazil). Both blood samples and *Leishmania* parasites were used for the preparation of standard curves (see below). Additionally, two non-engorged females of *Migonemyia migonei* obtained from a laboratory colony established in the Aggeu Magalhães Institute (Fiocruz-PE) were used as negative controls.

### Nucleic acid extraction

DNA extraction from sand flies, blood samples and *Leishmania* parasites was performed using DNeasy Blood & Tissue kit (Qiagen, Hilden, Germany), according to the manufacturer’s instructions. All samples were eluted in 100 µl of buffer AE (10 mM Tris Cl, 0.5 mM EDTA, pH 9.0), properly labelled and frozen at − 20 °C. The quantity and purity (absorbance ratio at 260/280 nm and at 260/230 nm) of the extracted DNA were accessed using a NanoDrop Lite spectrophotometer (Thermo Fisher Scientific, Waltham, USA).

### Primer and probe design

Primers and TaqMan hydrolysis probes (Table 1) targeting dog and human DNA were developed based on the sequences of the mitochondrial cytochrome *c* oxidase subunit 1 (*cox*1) and cytochrome *b* (*cytb*) genes, respectively, available from GenBank (accession numbers: NC_002008.4 and NC_012920.1), using the Primer3 v.0.4.0 (http://bioinfo.ut.ee/primer3-0.4.0/primer3/). Primers and probes were designed following the instructions of the TaqMan Multiplex Optimization User Guide [17] for optimum assay efficiency. In particular, primers should have a GC content of 40–60% and generate amplicons of 50–150 bp. The melting temperature (*T*_m_) should be similar for all primers; the *T*_m_ of the probes be ~ 10 °C higher than the *T*_m_ of the primers. With that in mind, primers (18–20 bp) and probes (13–25 bp) were designed to have *T*_m_ of 58–60 °C and 68–70 °C, respectively (Table 1).

**Table 1:** Primers and TaqMan probes used in the singleplex and multiplex real-time PCR assays

Species	Target	Primers and probes	Sequence (5′–3′)	Product size (bp)	References
*Canis familiaris*	*cox*1 gene	KF/CF-F (forward)	GGGGCTTTGGAAACTGACTA	95	Present study
		KF/CF-R (reverse)	TGGAGGAAGGAGTCAGAAGC		
		KF/CF-P (probe)	VIC-ATTGGTGCTCCGGACATGGCAT-QSY		
*Homo sapiens*	*cytb* gene	KF/HS-F (forward)	CCACCCTCACACGATTCTTT	104	Present study
		KF/HS-R (reverse)	GTTGTTTGATCCCGTTTCGT		
		KF/HS-P (probe)	NED-TGCAGCCCTAGCAACACTCCACC-NFQ-MGB		
*Leishmania* spp.	kDNA	LEISH-1 (forward)	AACTTTTCTGGTCCTCCGGGTAG	120	[18]
		LEISH-2 (reverse)	ACCCCCAGTTTCCCGCC		
		TaqMan-MGB (probe)	FAM-AAAAATGGGTGCAGAAAT-NFQ-MGB		

To avoid non-specific amplification, primers were submitted to BLAST/n of the National Centre for Biotechnology Information (NCBI) to verify its specificity. Furthermore, the formation of dimmers, hairpins, and *T*_m_ were assessed with the OligoAnalyzer 3.1 software (https://eu.idtdna.com/calc/analyzer). The primers LEISH-1 and LEISH-2 and a TaqMan probe (Table 1) were used to detect a 120 bp fragment of the *Leishmania* minicircle kinetoplast DNA (kDNA) [18].

### Optimization of singleplex real-time PCR assays

Before optimizing the multiplex real-time PCR assay, singleplex real-time PCR assays were optimized to specifically detect dog and human DNA. Additionally, a singleplex real-time PCR assay targeting kDNA was performed as described elsewhere [19]. A dilution matrix was made to determine optimal concentration of primers and probes [17]. The reaction mixture contained 1.35 µl of each primer (final concentration of 900 nM each), 0.3 µl of the probe (200 nM), 2.5 µl of water (DNAse and RNAse free), 7.5 µl of TaqMan Fast Advanced Master Mix (Thermo Fisher Scientific) and 2-µl sample DNA, in a final volume of 15 μl. Positive controls consisted DNA extracted from dog and human blood and from cultured promastigotes of *L. infantum*, whereas a master mix without DNA and DNA from unfed female sand flies were used as no template control (NTC) and negative control, respectively. The real-time PCR thermal conditions were as follows: 20 s at 95 °C followed by 35 cycles of 1 s at 95 °C and 20 s at 60 °C (estimated running time: 33.5 min). All singleplex real-time PCRs were performed on a QuantStudio 5 Real-Time PCR system (Thermo Fisher Scientific), with automatic baseline and threshold settings. The reactions were performed in triplicate, with inconsistent or undetermined results between the replicates being regarded as negative.

### Optimization of the multiplex real-time PCR assay

Sets of primers and probes targeting dog *cox1*, human *cytb* and *Leishmania* kDNA were multiplexed by labelling each probe with a different dye (Table 1). The reaction mixture consisted of 0.675 µl of each primer (900 nM), 0.15 µl of each probe (200 nM), 7.5 µl of TaqMan^®^ Fast Advanced Master Mix (Applied Biosystems) and 3-µl sample DNA, in a final volume of 15 μl. Positive controls consisted of mixed DNA extracted from dog and human blood and cultured promastigotes of *L. infantum*. Negative controls and thermal cycling conditions employed in the multiplex assay were the same used in the singleplex assays. All the three channels (reporter and quencher) for VIC-QSY, NED-NFQ/MGB and FAM-NFQ were selected. All multiplex real-time PCR assays were run on a QuantStudio 5 Real-Time PCR system (Thermo Fisher Scientific), with automatic baseline and threshold settings. The reactions were performed in triplicate and repeated three times, with inconsistent or undetermined results between the replicates being regarded as negative.

### Specificity, sensitivity, linearity and reproducibility

An *in silico* analysis of the specificity of the primers and probes was checked using program BLAST/n. The analytical specificity was assessed through cross-tests between the targets (dog, human, *L*. *braziliensis* and *L*. *infantum*) and unfed female sand flies. Standard curves were prepared using nine serial dilutions (10^8^, 10^7^, 10^6^, 10^5^, 10^4^, 10^3^, 10^2^, 10^1^ and 10^0^ fg per reaction) of DNA from dog, human and *L*. *infantum* to assess the analytical sensitivity (detection limit) of the assays. The analytical sensitivity was defined as the lowest amount of DNA detectable in a given assay. The cut-off point of an assay was defined as the quantification cycle (Cq) value corresponding to the detection limit [20].

The amplification efficiency (*E*) was calculated using the slope of the regression line in the standard curve through the equation: *E* = 10^(−1/slope)^ – 1. A slope close to − 3.33 was considered optimal. The correlation coefficient (*R*^2^) value was automatically calculated using measure of the closeness of fit between the regression line and the individual Cq data points of the standard reactions [21]. The y-intercept value also automatically calculated and corresponds to the theoretical Cq value for a single copy of the target molecule. In intra-assays, triplicates were made in the same plate, whereas in inter-assay, triplicates were repeated in three independent assays performed in three different days within a week.

### Assay of field-collected sand flies

A total of 97 engorged female sand flies collected in the context of a previous study [22] were tested individually by the newly developed multiplex real-time PCR. These females belonged to two species: *M. migonei* (*n* = 95); and *Nyssomyia intermedia* (*n* = 2). Details regarding sand fly collection, identification and processing are described elsewhere [22]. Females were collected both indoors (*n* = 23) and outdoors (*n* = 74).

### Data analysis

Real-time PCR results were analysed using QuantStudio Design and Analysis Software 1.3.1 (Thermo Fisher Scientific). To assess intra- and inter-assay reproducibility, the percent coefficient of variation (% CV) was calculated for each set of triplicate reactions. The positivity rates for *Leishmania* parasites in female sand flies collected indoors and outdoors was compared using Fisher’s exact test, considering *P* < 0.05 as statistically significant. Statistical analysis and calculations were performed using GraphPad Prism 5.0 software (GraphPad Software Inc., CA, USA).

## Results

### Specificity, sensitivity and linearity of the singleplex and multiplex assays

The sets of primers and probes specific for dog *cox*1, human *cytb* and *Leishmania* kDNA detected only the expected target. Moreover, they did not produce any non-specific amplification in the cross-testing with non-target DNA, or with no template and negative controls. Likewise, there were no false positives due to cross-talk between dye signals from each assay. Thus, analytical specificity of the assays was considered to be 100%.

The analytical sensitivity of the singleplex real-time PCR assays for dog *cox*1 and human *cytb* was 1000 fg, with Cq values of 34.2 ± 0.4 and 33.2 ± 0.1, respectively. Conversely, the analytical sensitivity for *Leishmania* kDNA was 1 fg (Cq = 33.5 ± 0.1) (Table 2).

**Table 2:** Analytical sensitivity and corresponding threshold cycle (Cq) values from singleplex and multiplex real-time PCR assays for each target

DNA sample	Quantity (fg/reaction)	Cq value (mean ± SD)	
		Singleplex	Multiplex
*Canis familiaris*	10^8^	17.92 ± 0.17	17.91 ± 0.15
	10^7^	21.14 ± 0.06	20.38 ± 0.99
	10^6^	24.43 ± 0.04	23.80 ± 0.04
	10^5^	27.79 ± 0.10	27.09 ± 0.20
	10^4^	31.09 ± 0.11	30.14 ± 0.19
	10^3^	34.21 ± 0.40	33.14 ± 1.03
*Homo sapiens*	10^8^	17.81 ± 0.05	16.11 ± 0.31
	10^7^	20.84 ± 0.12	18.34 ± 0.14
	10^6^	24.12 ± 0.04	21.73 ± 0.58
	10^5^	27.43 ± 0.08	25.31 ± 0.23
	10^4^	30.55 ± 0.24	27.90 ± 0.37
	10^3^	33.20 ± 0.07	30.59 ± 0.28
*Leishmania* spp.	10^8^	8.64 ± 0.05	9.18 ± 0.31
	10^7^	12.38 ± 0.10	11.43 ± 0.14
	10^6^	15.51 ± 0.12	14.61 ± 0.06
	10^5^	19.11 ± 0.04	17.88 ± 0.16
	10^4^	22.63 ± 0.07	21.45 ± 0.17
	10^3^	26.20 ± 0.02	24.44 ± 0.23
	10^2^	29.77 ± 0.13	27.46 ± 0.05
	10^1^	32.33 ± 0.23	30.88 ± 0.25
	10^0^	33.49 ± 0.09	33.11 ± 0.25

*Abbreviation*: SD, standard deviation

**Fig. 1:**
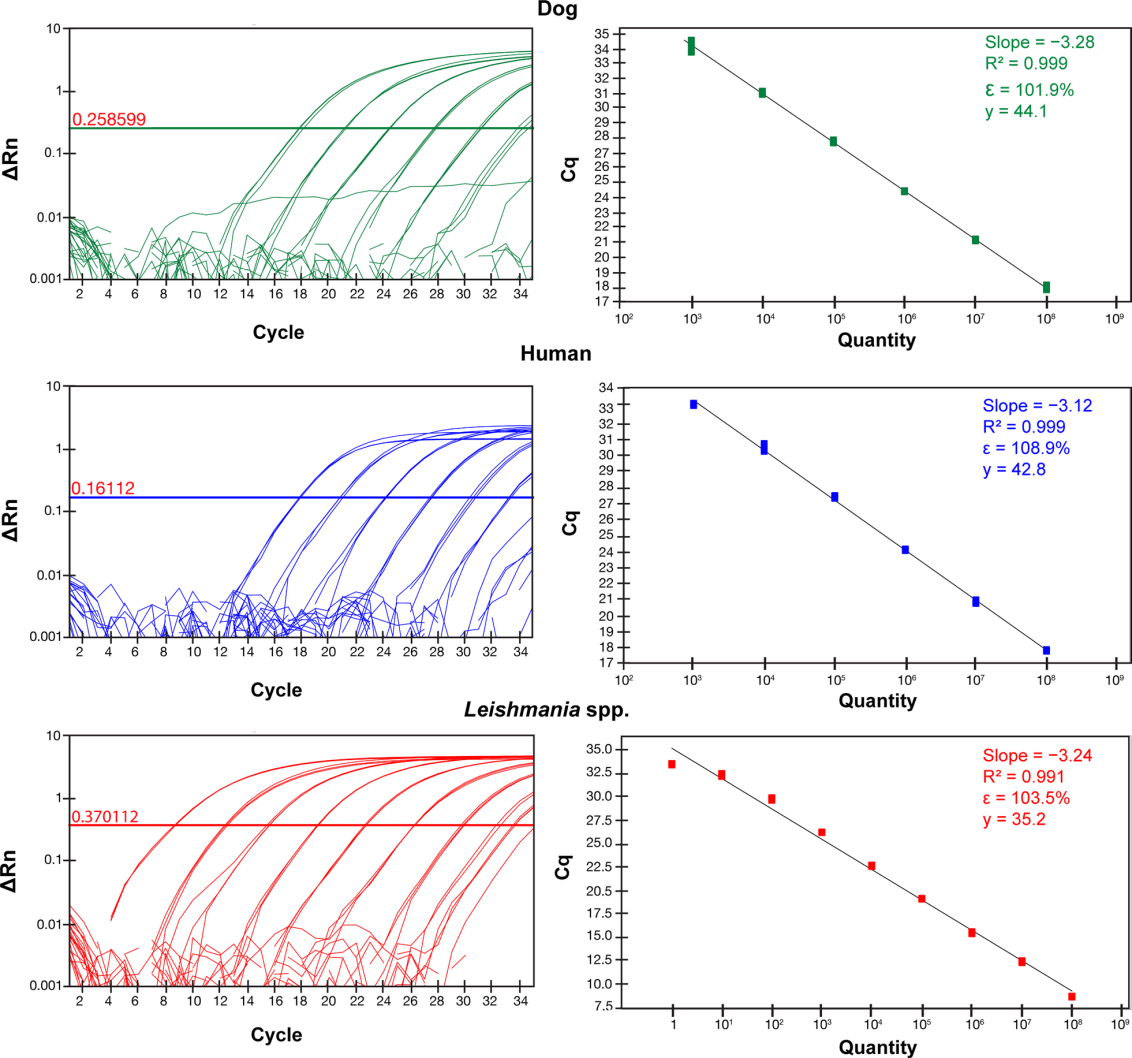
Amplification plots (left) and standard curves (right) of the singleplex real-time PCR assays, showing values of slope, correlation coefficient (*R*^2^), efficiency (*ε*) and y-intercept (y) for each target (dog *cox1*, human *cytb* and *Leishmania* kDNA). All DNA samples were tested in triplicate and curves below the threshold line are negative

The linear regression analysis of standard curves confirmed linearity of the singleplex real-time PCR assays for dog *cox*1 (*R*^2^ = 0.999, *E* = 101.9, slope = − 3.28, y-intercept = 44.1), human *cytb* (*R*^2^ = 0.999, *E* = 108.9, slope = − 3.12, y-intercept = 42.8) and *Leishmania* kDNA (*R*^2^ = 0.991, *E* = 103.5, slope = − 3.24, y-intercept = 35.2) (Fig. 1).

**Fig. 2:**
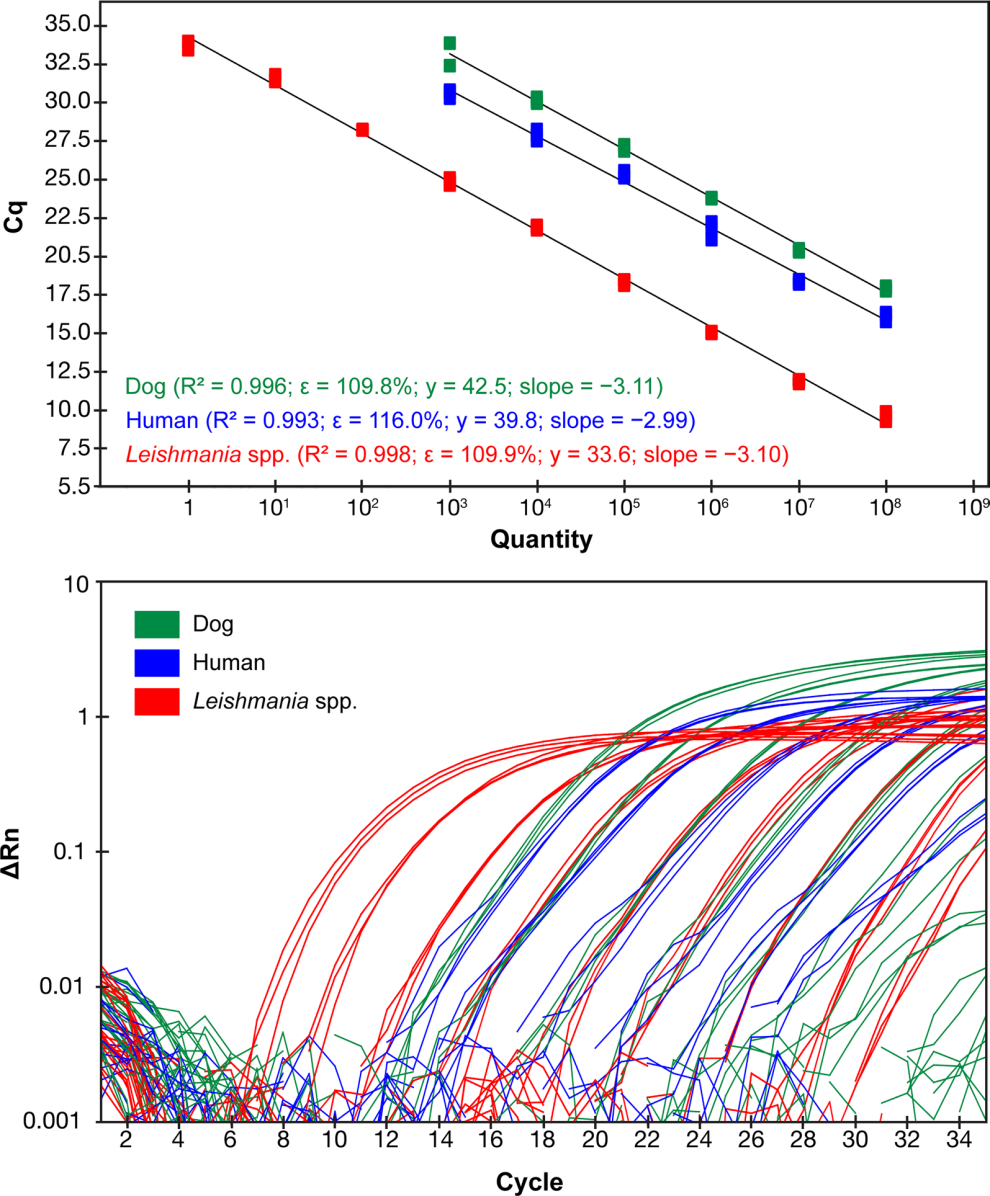
Standard curve (top) and amplification plot (bottom) of the multiplex real-time PCR assay, showing values of slope, correlation coefficient (*R*^2^), efficiency (*ε*) and y-intercept (y) for each target (dog, human and *Leishmania* DNA). All DNA samples were tested in triplicate

Similar results were found with the multiplex real-time PCR assay, the detection limits for dog *cox*1 and human *cytb* being 1000 fg, with Cq values of 33.1 ± 1.0 and 30.6 ± 0.3, respectively. The detection limit for *Leishmania* kDNA was 1 fg (Cq = 33.1 ± 0.3) (Table 2). Similarly, the linear regression analysis of standard curves confirmed linearity of the multiplex real-time PCR assay for dog *cox*1 (*R*^2^ = 0.996, *E* = 109.8, slope = − 3.11, y-intercept = 42.5), human *cytb* (*R*^2^ = 0.993, *E* = 116.0, slope = − 2.99, y-intercept = 39.8) and *Leishmania* kDNA (*R*^2^ = 0.998, *E* = 109.9, slope = − 3.10, y-intercept = 33.6) (Fig. 2).

### Reproducibility of multiplex real-time PCR assay

The variability of the multiplex real-time PCR assay was assessed between and within runs based on standard curves. The coefficients of variation values of intra- and inter- assays were in the range of 0.16–4.01% (Table 3) and 0.92–7.44% (Table 4), respectively. Overall, the difference between Cq values intra- and inter-assay was ≤ 2, suggesting that the multiplex real-time PCR assay is reliable.

**Table 3:** Intra-assay reproducibility of the multiplex real-time PCR assay

DNA sample	Quantity (fg/reaction)	Cq value			Mean ± SD	%CV
		R1	R2	R3		
*Canis familiaris*	10^8^	17.27	17.27	17.01	17.18 ± 0.15	0.87
	10^7^	19.23	19.17	19.30	19.24 ± 0.06	0.33
	10^6^	22.98	22.22	21.73	22.31 ± 0.63	2.82
	10^5^	25.06	25.44	26.14	25.55 ± 0.55	2.15
	10^4^	28.94	29.02	28.74	28.90 ± 0.15	0.51
	10^3^	33.61	32.99	34.48	33.69 ± 0.75	2.22
*Homo sapiens*	10^8^	16.67	16.99	16.87	16.84 ± 0.16	0.96
	10^7^	18.76	18.74	18.87	18.79 ± 0.07	0.39
	10^6^	22.50	22.35	22.74	22.53 ± 0.20	0.88
	10^5^	25.87	25.57	25.72	25.72 ± 0.15	0.58
	10^4^	28.48	28.52	27.83	28.27 ± 0.38	1.36
	10^3^	30.94	33.24	33.13	32.44 ± 1.30	4.01
*Leishmania* spp.	10^8^	5.96	5.92	6.06	5.98 ± 0.07	1.23
	10^7^	8.58	8.87	9.16	8.87 ± 0.29	3.28
	10^6^	12.54	12.22	12.56	12.44 ± 0.19	1.53
	10^5^	15.81	15.58	16.32	15.91 ± 0.38	2.39
	10^4^	19.31	19.43	19.78	19.51 ± 0.25	1.26
	10^3^	23.16	23.06	22.72	22.98 ± 0.23	1.02
	10^2^	26.66	26.21	26.78	26.55 ± 0.30	1.13
	10^1^	29.82	29.91	29.83	29.85 ± 0.05	0.16
	10^0^	32.03	32.10	32.52	32.22 ± 0.26	0.81

*Abbreviations*: Cq, quantification cycle; R, replicate; SD, standard deviation; %CV, percent coefficient of variation

**Table 4:** Inter-assay reproducibility of the multiplex real-time PCR assay

DNA sample	Quantity (fg/reaction)	Cq value (mean)			Mean ± SD	%CV
		D1	D2	D3		
*Canis familiaris*	10^8^	17.18	17.61	17.29	17.36 ± 0.23	1.30
	10^7^	19.24	19.80	19.38	19.47 ± 0.29	1.51
	10^6^	22.31	23.30	22.80	22.8 ± 0.50	2.17
	10^5^	25.55	26.62	26.18	26.12 ± 0.54	2.07
	10^4^	28.90	29.95	29.57	29.47 ± 0.53	1.80
	10^3^	33.69	34.42	33.54	33.88 ± 0.47	1.39
*Homo sapiens*	10^8^	16.84	15.51	16.43	16.26 ± 0.68	4.19
	10^7^	18.79	17.57	18.18	18.18 ± 0.61	3.35
	10^6^	22.53	21.30	21.86	21.90 ± 0.62	2.82
	10^5^	25.72	24.95	25.33	25.33 ± 0.39	1.53
	10^4^	28.27	27.65	27.78	27.90 ± 0.33	1.18
	10^3^	32.44	32.21	31.78	32.14 ± 0.34	1.04
*Leishmania* spp.	10^8^	5.98	6.76	6.86	6.53 ± 0.48	7.37
	10^7^	8.87	9.95	10.24	9.68 ± 0.72	7.44
	10^6^	12.44	13.48	13.77	13.23 ± 0.70	5.28
	10^5^	15.91	16.91	17.47	16.76 ± 0.79	4.73
	10^4^	19.51	20.50	20.87	20.29 ± 0.70	3.47
	10^3^	22.98	24.02	24.84	23.95 ± 0.93	3.89
	10^2^	26.55	27.43	25.47	26.48 ± 0.98	3.71
	10^1^	29.85	30.40	30.33	30.20 ± 0.30	0.99
	10^0^	32.22	32.37	32.79	32.46 ± 0.30	0.92

*Notes*: In each day, DNA samples were tested in triplicate (mean values reported)

*Abbreviations*: Cq, quantification cycle; D, day; SD, standard deviation; %CV, percent coefficient of variation

### Evaluation of multiplex real-time PCR assay with field-collected sand flies

Fifty out of 97 (51.6%) engorged female sand flies tested by the multiplex real-time PCR assay were positive. All positive females belonged to the species *M*. *migonei.*

Forty (80.0%) females were positive for one target (i.e. human *cytb*, dog *cox*1 or *Leishmania* kDNA) and 10 (20.0%) for two targets. Among females that were positive for one target (*n* = 40), 85.0% were positive for human *cytb*, 10.0% for *Leishmania* kDNA and 5.0% for dog *cox*1. Among those positive for two targets (*n* = 10), 80.0% were simultaneously positive for human *cytb* and *Leishmania* kDNA and 20.0% were simultaneously positive for human *cytb* and dog *cox*1.

Out of 12 *Leishmania*-positive females, eight were fed on humans and none were fed on dogs. Four positive females were collected indoors and eight outdoors (Fisher’s exact test, *P* = 0.4704).

## Discussion

In this study, we were interested in developing a tool that could generate information about role of sand flies collected inside human houses and surrounding areas in the transmission of *Leishmania* parasites to dogs and humans. Hence, we developed a TaqMan-based fast multiplex quantitative real-time PCR assay for the simultaneous detection of dog *cox1*, human *cytb* and *Leishmania* kDNA in female sand flies. The addition of multiple primers and probes in a single reaction as well as changes in the number of cycles and annealing temperature can affect the specificity, sensitivity and efficiency of real-time PCR assays [23, 24]. This is in fact one of the main obstacles to overcome while developing a multiplex real-time PCR assay [17]. Although many singleplex assays have been successful in identifying blood meal and *Leishmania* parasites in sand flies [12–15, 25], none of them combined the detection of different host and the parasite DNA in a one-step assay. The development of the multiplex real-time PCR assay proposed by the present study resulted in a series of advantages compared to other assays, such as the reduction in reagent consumption, labour time and the ability to provide faster results (considering that regular real-time PCR assays take over 1 hour to complete, e.g. ~ 90 min [12, 18], ~ 78 min [13]). In practice, this assay allows testing a great number of sand flies for both dog *cox1*, human *cytb* and *Leishmania* kDNA in a shorter period of time (~ 34 min), reducing overall costs.

Considering that female sand flies consume a small amount of blood (≤ 1 μl) during blood-feeding [26], one of the main technical challenges while developing a molecular tool for detecting host blood and *Leishmania* parasites is the necessity to detect and quantify a very limited amount of DNA [16]. In fact, the sensitivity of such an assay depends directly on both the initial quantity of the target DNA in the sample and on the time span from blood ingestion, as there is a progressive degradation of the host DNA during blood digestion [12, 16, 27]. The multiplex real-time PCR assay developed in this study was shown to be specific and highly sensitive, without interference and competition between targets and dyes. Particularly, identical analytical sensitivities were obtained with singleplex and multiplex assays (i.e. 10^3^ fg/reaction for dog *cox*1 and human *cytb*, and 1 fg for *Leishmania* kDNA). These results are similar to those obtained with other assays for detecting blood meals of female sand flies, which reported a detection limit from 10^2^ fg to 10^4^ fg of host DNA [6, 12, 27]. More recently, two SYBR Green-based real-time PCR assays were reported to have a detection limit of 26 fg for dog and 84 fg for human [13]. Despite the good analytical sensitivity of these assays, when non-target DNA samples were used some slight noise was reported in the melting curve analysis, though with a Cq value always higher than 30 [13]. In fact, non-specific signals are a known limiting factor of some SYBR Green-based real-time PCR assays [12, 28], which may eventually obscure the interpretation of the results. The use of hydrolysis probes (e.g. TaqMan probes) may increase the specificity of real-time PCR assays as demonstrated elsewhere [29, 30].

For *Leishmania* kDNA detection, the analytical sensitivity of our multiplex real-time PCR was similar to a singleplex assay using the same primers and probe [19], allowing the detection of less than a single parasite per sample. This high sensitivity may be partly attributed to the target used (i.e. kDNA), which is present in high number of copies (~ 10,000 copies of minicircle molecules) per parasite [31]. In fact, other real-time PCR assays using the same target gene also reported very good analytical sensitivity [32, 33].

Our multiplex real-time PCR assay was also successfully applied in field-collected samples. A total of 44 females of *M. migonei* (stored at − 20 °C for ~ 2 years) were positive for human blood. This sand fly species displays a remarkable degree of anthropophily, and it is a proven vector of *L. braziliensis* and a putative vector of *L. infantum* in Latin America [34, 35]. Interestingly, eight out of 44 females fed on humans were also positive for *Leishmania* spp. *Leishmania*-positive sand flies were collected in human dwellings (four indoors and eight outdoors), where human cases of CL by *L. braziliensis* were previously recorded [22]. Altogether, these findings strongly suggest that *M. migonei* is a vector of *L. braziliensis* for humans in the indigenous villages, where sand flies were collected. While our multiplex real-time PCR assay was tested with sand flies collected from a CL focus, it is also suitable for other epidemiological settings, namely VL endemic regions, considering that the primers and probe used also efficiently detected *L. infantum* kDNA [18, 19].

The identification of humans as the most frequent host of *M. migonei* females in this study, also reinforces their high attractiveness for humans [34] as well as the hypothesis that this species may be adapted to feed indoors [22]. It has been shown that human CL patients that have been treated and clinically cured may harbour viable parasites in their scars [36] and it has been suggested that they could eventually act as a source of infection to sand flies [37]. Our multiplex real-time PCR assay may be a useful tool to assess the presence of *Leishmania* parasites and human blood in sand flies from other CL-endemic areas, ultimately to investigate their possible role in the transmission cycle of *L. braziliensis*.

The finding of eight *Leishmania*-positive females which previously fed on humans raises interesting questions regarding whether these females acquired the infection from a previous unknown host (e.g. small rodents) [38] or from humans themselves. Interestingly, four *Leishmania*-positive engorged female sand flies did not apparently feed on humans or dogs, further suggesting that they probably acquired the parasites from another host.

## Conclusions

In conclusion, a novel TaqMan-based fast multiplex real-time PCR assay was developed, optimized and validated herein for simultaneous detection of dog and human blood meals and *Leishmania* parasites in female sand flies. This assay may represent a tool for assessing *Leishmania* parasite infection in female sand flies and for investigating whether and how often these females feed on dogs and humans, thereby allowing estimation of the risk of infection in these hosts.

## Data Availability

The data supporting the conclusions of this article are included within the article. Raw data can be shared with other researchers upon a specific request.

## Abbreviations

VL: visceral leishmaniasis
CL: cutaneous leishmaniasis
PCR: polymerase chain reaction
ELISA: enzyme-linked immunosorbent assay
EDTA: ethylene diamine tetra acetic acid
DNA: deoxyribonucleic acid
kDNA: kinetoplast minicircle DNA
*cox*1: mitochondrial cytochrome *c* oxidase subunit 1 gene
*cytb*: cytochrome *b* gene
GC: guanine/cytosine
BLAST: Basic Local Alignment Search Tool
NCBI: National Center for Biotechnology Information
NTC: no template control
Tm: melting temperature
Cq: quantification cycle
ΔRn: delta Rn
E: efficiency
%CV: percent coefficient of variation
SD: standard deviation
